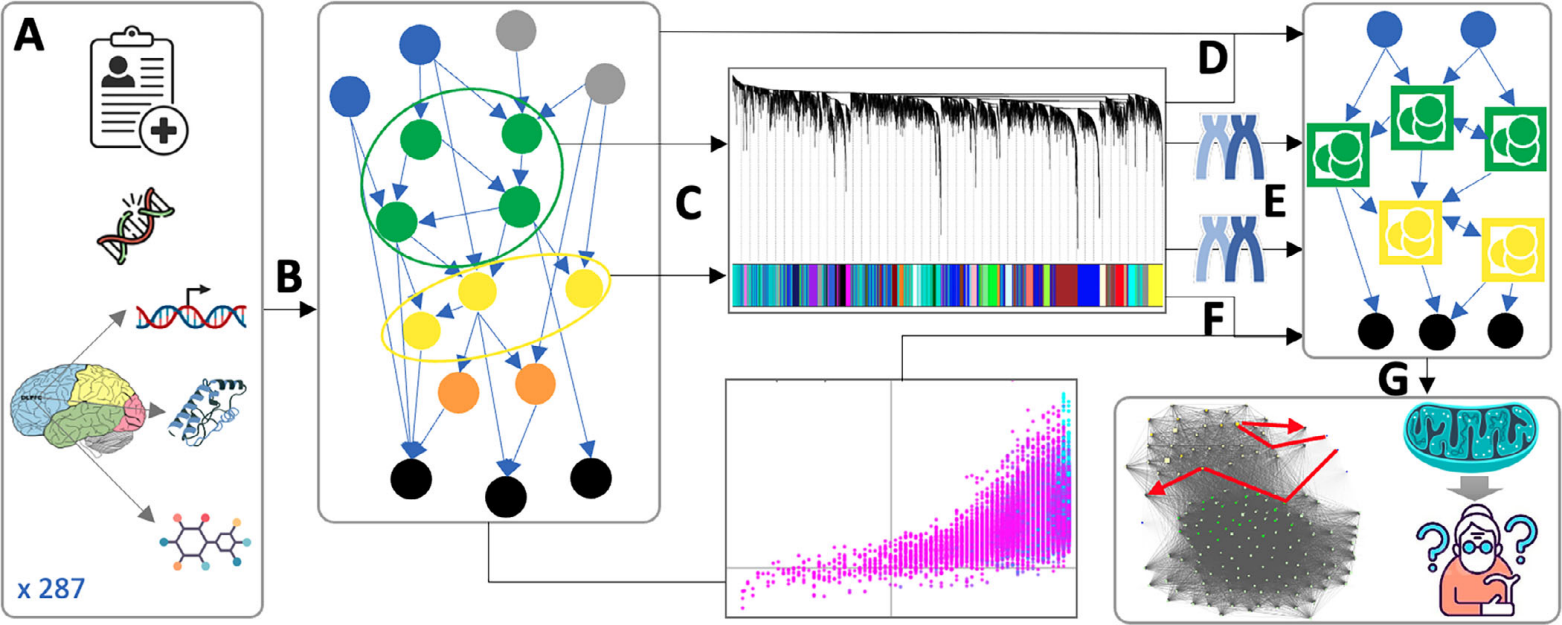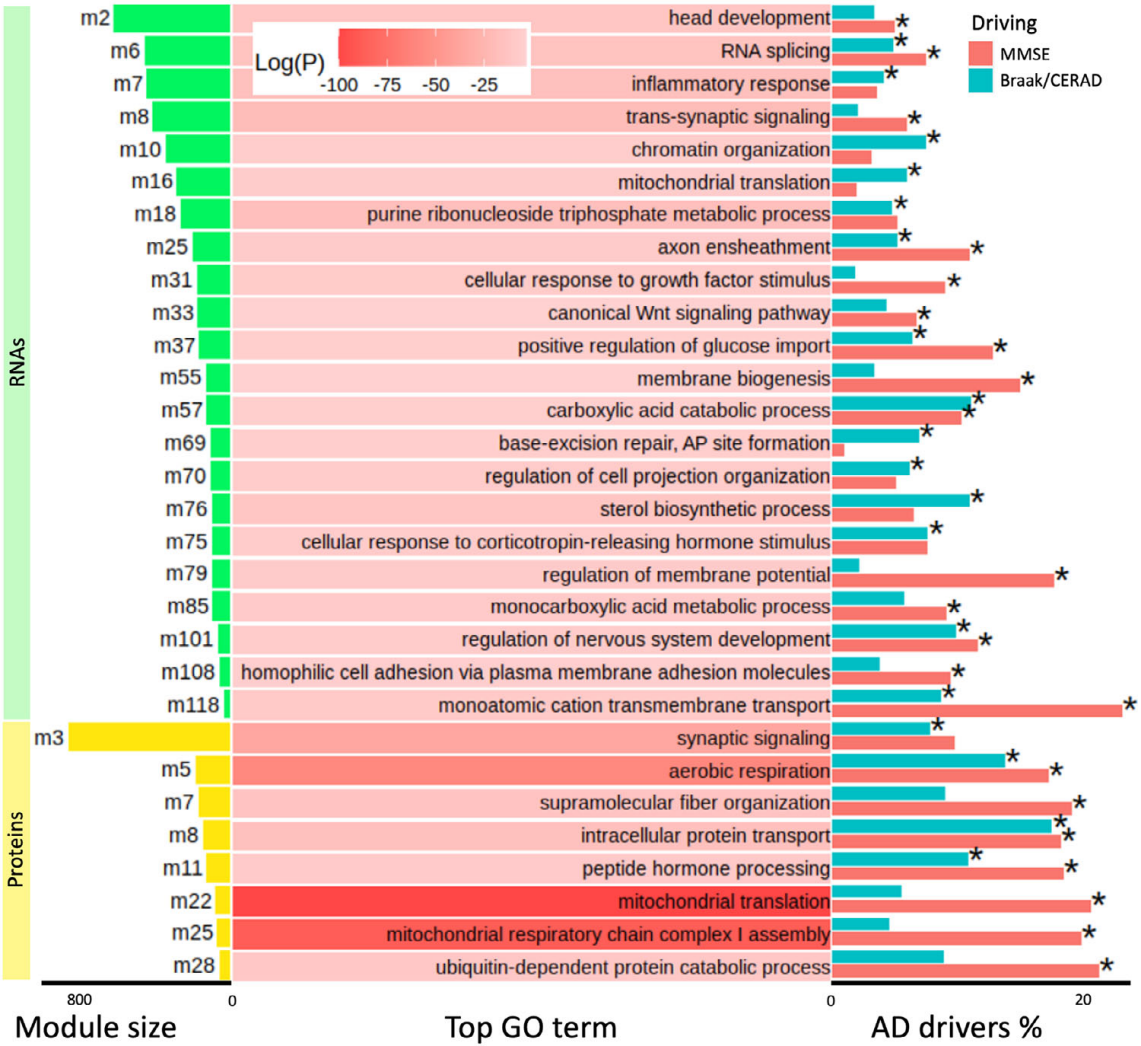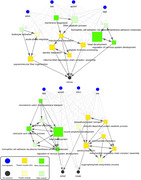# Causal network analysis on multi‐omics data from Alzheimer’s brain reveals strong connection between mitochondria functions and AD outcomes

**DOI:** 10.1002/alz.085208

**Published:** 2025-01-03

**Authors:** Li Sun, Apoorva Bharthur Sanjay, Ole Isacson, So‐Youn Shin, Jeanne Latourelle

**Affiliations:** ^1^ Aitia, Somerville, MA USA; ^2^ Harvard Medical School / McLean Hospital, Belmont, MA USA

## Abstract

**Background:**

Alzheimer’s disease (AD) is a progressive neurodegenerative disease and the most prevalent type of senile dementia affecting more than 6 million Americans in 2023. Most of these AD cases are sporadic or late‐onset AD with unclear etiology. Recent clinical trials on antibody drug clearing Ab plagues in brain show modest benefits of slowing down cognitive decline. This indicates that a deeper and more comprehensive understanding of AD mechanism is needed for better therapeutic developments.

**Method:**

Using Aitia’s proprietary REFS^TM^ platform, Bayesian network models were built on clinical, demographic, and multi‐omic data from 287 postmortem brain samples of ROSMAP project. *In silico* perturbations were performed to assess causal effects among the variables. Hierarchical clustering was conducted to identify RNA‐ and protein‐ modules and to summarize a module‐level causal network, where a module‐level causal effect was computed as summated causal effects normalized to module sizes. For each module, gene ontology (GO) terms were annotated using Metascape.

**Result:**

A total of 119 RNA‐ and 33 protein‐ modules were identified, of which 22 RNA‐ and 8 protein‐ modules were enriched with top genes driving AD outcomes identified through *in silico* perturbations. These modules were significantly over‐presented with various GO terms. Notably, three out of eight protein‐ modules are strongly related to mitochondria functions. Moreover, three pairs of RNA‐ and protein‐ modules identified for their similar enrichment patterns are related to synaptic signaling & neuron development, mitochondria translation, and complex I assembly.

The module‐level causal network highlighted that the paths from AD risk factors (e.g. age, sex, APOE4) to AD outcomes (e.g. MMSE, Braak, Cerad) were strongly connected through three protein modules annotated for mitochondria function and three RNA modules annotated for nervous system development, while the paths to the cognitive outcome only (e.g. MMSE) included two immune response related and one cytoskeleton related modules as well.

**Conclusion:**

Clustering analysis based on *in silico* causal relationships of clinical and multi‐omic data revealed a strong connection of genes involved in mitochondria functions with cognition and AD pathology, reminiscing “mitochondria cascade hypothesis” of AD first proposed two decades ago.